# Salicylic acid and reactive oxygen species interplay in the transcriptional control of defense genes expression

**DOI:** 10.3389/fpls.2015.00171

**Published:** 2015-03-19

**Authors:** Ariel Herrera-Vásquez, Paula Salinas, Loreto Holuigue

**Affiliations:** Departamento de Genética Molecular y Microbiología, Facultad de Ciencias Biológicas, Pontificia Universidad Católica de ChileSantiago, Chile

**Keywords:** glutathione, glutaredoxin GRXC9/GRX480, NPR1, reactive oxygen species, salicylic acid, thioredoxin TRXh5, TGA transcription factors

## Abstract

It is well established that salicylic acid (SA) plays a critical role in the transcriptional reprograming that occurs during the plant defense response against biotic and abiotic stress. In the course of the defense response, the transcription of different sets of defense genes is controlled in a spatio-temporal manner via SA-mediated mechanisms. Interestingly, different lines of evidence indicate that SA interplays with reactive oxygen species (ROS) and glutathione (GSH) in stressed plants. In this review we focus on the evidence that links SA, ROS, and GSH signals to the transcriptional control of defense genes. We discuss how redox modifications of regulators and co-regulators involved in SA-mediated transcriptional responses control the temporal patterns of gene expression in response to stress. Finally, we examine how these redox sensors are coordinated with the dynamics of cellular redox changes occurring in the defense response to biotic and abiotic stress.

## Interplay between Salicylic Acid (SA) and Redox Signals in the Defense Response to Stress

**FIGURE 1:**
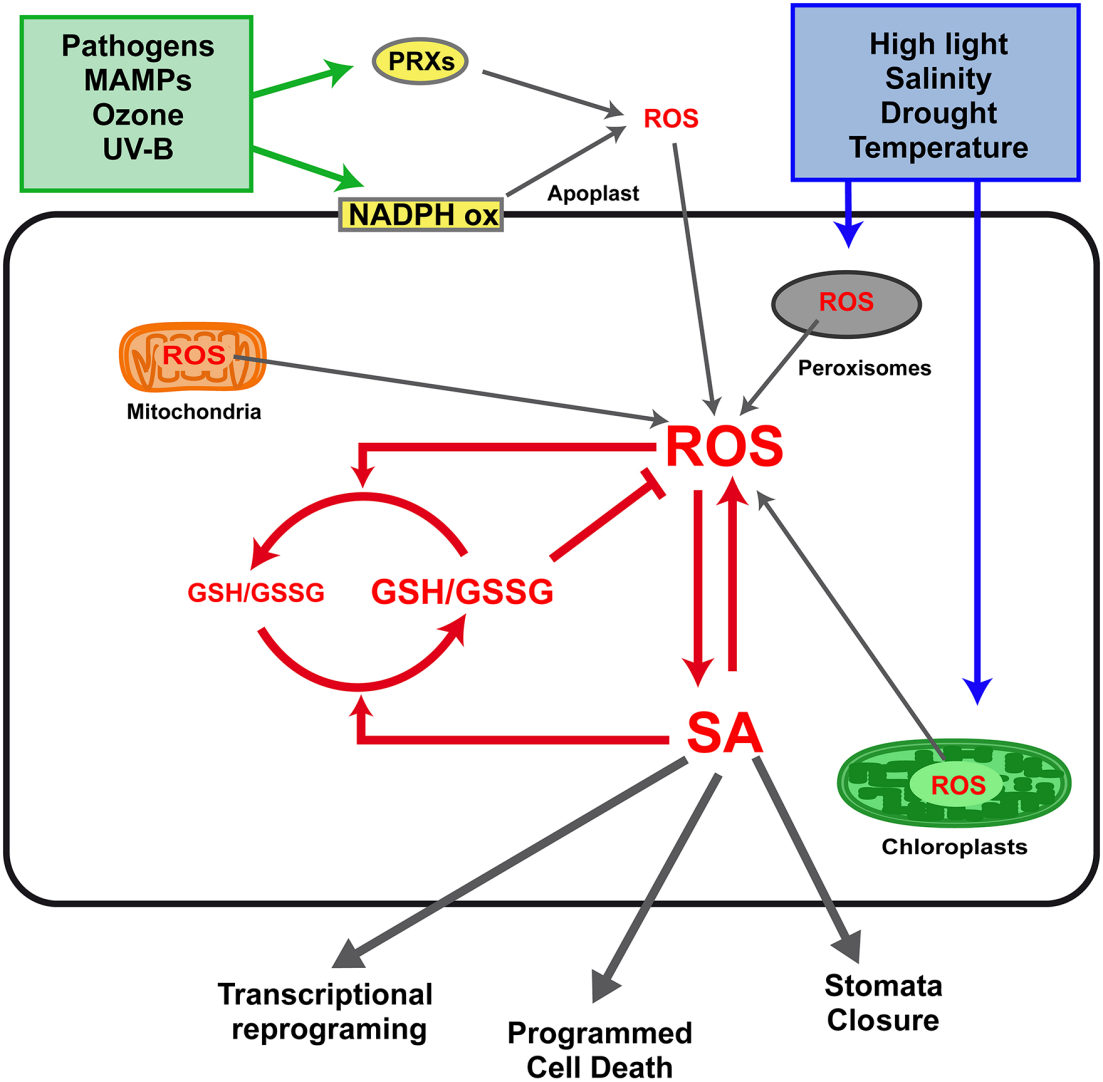
Interplay between salicylic acid (SA), reactive oxygen species (ROS), and glutathione (GSH) in defense responses to biotic and abiotic stress. Stress conditions such as infection with pathogens, exposure to microbe-associated molecular patterns (MAMPs), ozone, and UV-B treatments, trigger ROS production mainly at the apoplast. This production of ROS is mediated by plasma membrane NADPH oxidases (NADPH ox) and cell wall peroxidases (PRXs). Other stresses, such as high light radiation, salinity, drought, and temperature, trigger ROS production mainly at the chloroplasts and peroxisomes. Mitochondria have been also described as an important source of ROS during defense responses (Lam et al., 2001). A feed-forward loop between H_2_O_2_ and SA synthesis occurs in response to stress, as described in the text. SA also has an antioxidant role, increasing GSH levels and reducing power, which in turn is involved in ROS scavenging. Finally, the interplay between intracellular levels of SA, H_2_O_2_, and GSH determines transcriptional reprogramming, programmed cell death, and stomata closure, the three main outputs of the defense responses.

A feed-forward loop between salicylic acid (SA) and reactive oxygen species (ROS) production in the defense response to stress was first reported at the early 1990s (Chen et al., 1993). This early report was followed by a controversy on whether H_2_O_2_ was downstream or upstream of SA in the pathway for induction of *Pathogenesis-Related 1* (*PR1*) expression (Neuenschwander et al., 1995; Chamnongpol et al., 1996). Later on, it was demonstrated that ROS signals are involved both upstream and downstream SA signaling in response to stress. Interestingly, the evidence indicates that SA does not only play a pro-oxidant role, but it also has an antioxidant role in concert with glutathione (GSH) in the response to stress. In this first section we present a comprehensive picture of the relationships between SA, ROS, and GSH in the response to stress signaling (**Figure 1**).

### ROS Bursts Trigger SA Signaling

It is well known that activation of SA signaling in stressed plants is preceded by oxidative bursts originating in different cellular compartments (Wrzaczek et al., 2013). In the case of basal (PTI) and induced (ETI) defense responses against pathogens infection, it has been extensively reported that increases in SA levels are preceded by apoplastic H_2_O_2_ bursts mediated by NADPH oxidases and extracellular peroxidases (PRXs; Mackerness et al., 2001; Torres et al., 2002; Joo et al., 2005; Tsuda et al., 2008; O’Brien et al., 2012; Mammarella et al., 2014). Although PTI and ETI responses are activated in the plant by recognition of different pathogens molecules, they share several signals including ROS and SA. Differences in the timing and levels at which these signals are produced in PTI and ETI determine differences in the speed and strength at which these immune reactions are established to be effective in counteracting potential pathogens with low cost on fitness (Tsuda et al., 2008; Katagiri and Tsuda, 2010).

Apoplastic H_2_O_2_ bursts also precede SA signaling in plant responses to exposure to ozone and UV-B (Grant and Loake, 2000; Mackerness et al., 2001; Torres et al., 2002; Joo et al., 2005; Ogawa et al., 2007; Garcion et al., 2008; O’Brien et al., 2012). Pharmacological evidences supports that increases in apoplastic H_2_O_2_ levels after UV-B trigger SA biosynthesis (Mackerness et al., 2001). Noteworthy, in the case of ozone, ROS signaling starts at guard-cells chloroplasts and then it propagates to the apoplast of neighbor cells (Joo et al., 2005).

Salicylic acid also functions as a signal of other types of abiotic stresses such as high light exposure, salinity, drought, and temperature (Mateo et al., 2006; Lee and Park, 2010; Wan et al., 2012; Miura and Tada, 2014). In contrast to the above mentioned stresses, these conditions generate ROS accumulation in chloroplasts and peroxisomes (Apel and Hirt, 2004; Holuigue et al., 2007). Although involvement of SA in these cases has been demonstrated in SA-deficient and overproducer plants (Mateo et al., 2006; Lee and Park, 2010; Wan et al., 2012; Miura and Tada, 2014), direct evidence of increased SA levels has been only reported in oat plants exposed to drought (Sánchez-Martín et al., 2014). Interestingly, increased levels of SA have been detected in plants with sustained ROS production in peroxisomes (catalase 2 knockout, *cat2*; Chaouch et al., 2010) and in chloroplast (thylakoidal ascorbate PRX gene silencing, *tAPX RNAi*; Maruta et al., 2012; Noshi et al., 2012). The evidence obtained using these models indicate that H_2_O_2_ originated in chloroplasts and peroxisomes triggers SA biosynthesis, which is essential for main outputs of the defense response: transcriptional reprogramming, cell death, and stomatal closure (**Figure 1**).

The mechanisms by which H_2_O_2_ generated in the apoplast, chloroplasts, and peroxisomes triggers SA biosynthesis remains unknown. *ICS1* and *ICS2* are the two *Arabidopsis* genes coding for isochorismate synthase, the key enzyme controlling SA biosynthesis (Garcion et al., 2008). *ICS1* upregulation was detected in the ETI response to pathogens, in response to UV-B, ozone, and drought stress (Wildermuth et al., 2001; Ogawa et al., 2007; Zhang et al., 2010; Wan et al., 2012), as well as in *cat2* plants (Chaouch et al., 2010). In contrast, upregulation of *ICS2* but not of *ICS1* was detected in *tAPX RNAi* plants (Noshi et al., 2012). Transcription factors that regulate *ICS1* expression, such as CBP60, SARD1, and WRKY8/28/48 (Zhang et al., 2010; van Verk et al., 2011; Gao et al., 2013), or upstream *PAD4/EDS1* genes expression, such as CAMTA3/SR1 and ZAT6 (Du et al., 2009; Shi et al., 2014) represent potential candidates for ROS-mediated regulation of SA biosynthesis.

Remarkably, it has recently been proposed that Ca^+2^ signaling regulate SA production (Seyfferth and Tsuda, 2014), based on evidence that the activity of CBP60, WRKY8/28/48, and CAMTA3/SR1 factors are modulated by calcium dependent protein kinases (CDPKs) and calmodulin (CaM; Du et al., 2009; Gao et al., 2013; Truman et al., 2013). Indeed, intracellular increase of cytosolic Ca^+2^ was first described as an upstream signal that controls apoplastic ROS production through the modification of NADPH oxidase by CDPKs (Dubiella et al., 2013; Gao et al., 2013). Recently, Ca^+2^ has been also proposed to act downstream ROS signaling (Wrzaczek et al., 2013), based on previous evidence that exogenous treatments with H_2_O_2_ promote Ca^+2^ influxes (Price et al., 1994; Pei et al., 2000). Therefore, the possibility that a Ca^+2^ signal mediates activation of SA production triggered by ROS, represents an interesting aspect to explore.

### SA Modulates Redox Homeostasis

An ambivalent effect of SA in promoting ROS accumulation (prooxidant) and ROS scavenging (antioxidant), has being reported in several stress models, including the ETI response to pathogens and responses to high light, drought, salinity, and cold stress (Mou et al., 2003; Mateo et al., 2006; Miura and Tada, 2014). On one hand, SA promotes ROS production during early events of signaling, being these ROS essential for defense responses (Garreton et al., 2002; Lee et al., 2010; Khokon et al., 2011). Furthermore, high concentrations of SA (>100 μM) promote ROS production, inducing oxidative stress, and reducing tolerance to drought and salinity (Lee et al., 2010; Miura and Tada, 2014). How can SA promote ROS accumulation? Early reports showed SA-mediated inhibition of catalase and cytosolic ascorbate PRX, two main H_2_O_2_ detoxifying enzymes (Chen et al., 1993; Durner and Klessig, 1995). Then, SA-promoted production of ROS by extracellular PRXs was identified in stomatal closure control in drought response (Khokon et al., 2011; Miura et al., 2013).

On the contrary, the available evidence supports that SA promotes ROS scavenging being essential for the antioxidant response that constrains ROS bursts in responses to avirulent bacteria (Grant and Loake, 2000), high light (Mateo et al., 2006), ozone (Yoshida et al., 2009), salinity (Lee et al., 2010), and in *cat2* mutants (Chaouch et al., 2010). Recent studies show that SA and GSH interplay as redox signals, fostering a role for SA in the antioxidant response (Dubreuil-Maurizi et al., 2011; Foyer and Noctor, 2011; Dubreuil-Maurizi and Poinssot, 2012; Han et al., 2013). Plants that over accumulate SA show increased GSH levels and reducing power (ratio GSH/GSSG; Mateo et al., 2006) while abolishment of SA accumulation in a *cat2* background (*cat2 sid2*) reduces the GSH/GSSG ratio (Chaouch et al., 2010; Noctor et al., 2014). Conversely, plants deficient in GSH biosynthesis (phytoalexin-deficient mutant, *pad2-1*) have decreased levels of SA and* ICS1* transcripts (Dubreuil-Maurizi et al., 2011). This suggests that SA can play an antioxidant role by modulating GSH levels and reducing power (**Figure 1**), through still unknown mechanisms.

The dual redox effect of SA is reflected by a biphasic redox dynamics in plants treated with SA or INA (Mou et al., 2003; Mateo et al., 2006). A first oxidative phase, characterized by a transient increase in ROS levels and decline in GSH reducing power, is followed by a reductive phase characterized by an increase in GSH levels and reducing power. This temporal dynamics determines a sequential activation of the redox-regulated processes involved in the transcription of defense genes.

## Redox-Modulated Processes in the SA-Mediated Control of Gene Expression

**FIGURE 2:**
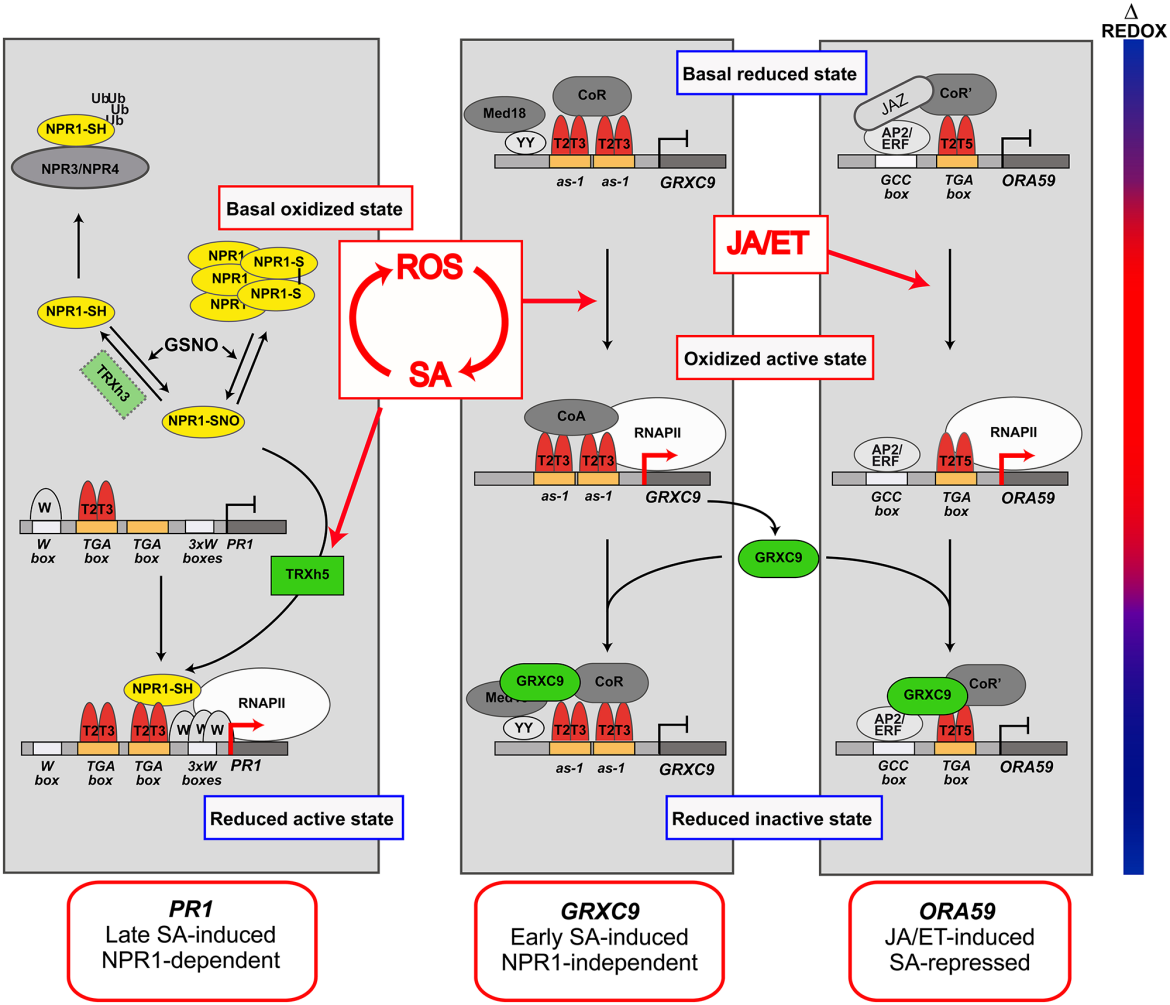
Redox-modulated processes in the SA-mediated control of gene expression. Model for the transcriptional control of genes representing three main groups of SA-regulated genes: SA-induced non-expressor of pathogenesis-related (PR) genes 1 (NPR1) -dependent late genes (*PR1*, **Left**); SA-induced NPR1-independent early genes [*glutaredoxin C9* (*GRXC9*), **Medium**]; and JA/ET-induced SA-repressed genes [*Octadecanoid-Responsive AP2/ERF domain protein 59* (*ORA59*), **Right**]. The temporal dynamics of the redox changes (Δ Redox) occurring during the defense response to stress are represented by the bar at the left, where blue indicates reductive states and red indicates oxidative states. The temporal dynamics in the formation of transcriptionally active and inactive complexes in the promoter of *PR1*, *GRXC9*, and *ORA59*, according to redox changes dynamics, are included in each panel. The places where ROS/SA, and JA/ET signals act in these pathways, is indicated by red arrows. The components identified (or suspected) as redox sensors in these pathways, whose mechanisms of action are discussed in the text, are indicated in color. TGA factors (red) are involved in the three pathways. Homodimers or heterodimers of TGA2 and TGA3 (T2T3) or TGA2 and TGA5 (T2T5) factors act as platforms for the formation of transcriptionally inactive and active complexes. Active complexes promote recruitment of RNA polymerase II (RNAPII) and gene transcription (red arrows at promoters). NPR1 (yellow) is the master co-activator for SA-inducible NPR1-dependent pathway and is redox-regulated by oxido-reduction of Cys residues. TRXh5 and GRXC9 (green) are oxidoreductases coded by SA-inducible genes, which catalyze reduction of NPR1 and of a still unknown component in *GRXC9* and *ORA59* promoters. Other transcriptional factors and co-factors not directly involved in redox regulation are shown in gray tones.

Salicylic acid plays a pivotal role in the genetic reprogramming, being responsible for transcriptional control of 100s of defense genes that are sequentially turned on/off (Maleck et al., 2000; Wang et al., 2006; Blanco et al., 2009). Interestingly, several redox-regulated processes have been discovered in the transcription of SA-regulated genes (Mou et al., 2003; Koornneef et al., 2008; Tada et al., 2008). The evidence suggests that cellular redox changes occurring in response to stress are translated into transcriptional responses, through redox modifications of master regulators and co-regulators (Moore et al., 2011). Here, we focus in the redox-modulated processes mediated by SA that control the expression of three *Arabidopsis* model genes: *PR1*, *GRXC9* (*glutaredoxin C9* or *GRX480*), and *ORA59* (*Octadecanoid-Responsive AP2/ERF domain protein 59*; **Figure 2**). These genes have been studied in greater detail and they respond to SA with particular temporal patterns and mechanisms, being therefore good models for different classes of SA-regulated genes.

Members of the TGA and WRKY transcription factor families, that recognize the TGA box (TGACGTCA) and the W box (TTGACT), respectively, have been involved in SA-mediated transcriptional regulation (Pandey and Somssich, 2009; Gatz, 2013). Furthermore, co-regulators including non-expressor of *PR* genes 1 (NPR1), SCL14, and Med 18 control transcription of different groups of SA-regulated genes (Fu and Dong, 2013). In this second section we will focus our attention on NPR1, TGA factors, and two oxidoreductases, to discuss evidence that point them as redox sensors in the expression of SA-regulated genes (**Figure 2**).

### Non-Expressor of *PR G*enes 1 (NPR1), a Master Redox Sensor

Non-Expressor of *PR* genes is the master co-activator for *PR1* and most SA-induced genes, and was the first redox sensor described for SA-regulated genes (Mou et al., 2003). Particularly at the *PR1* promoter, SA stimulates NPR1 interaction with TGA2 and TGA3, which enhances its binding to TGA boxes, forming a *trans*-activating complex for RNA polymerase II (RNAPII) recruitment (**Figure 2**, left panel; Lebel et al., 1998; Kesarwani et al., 2007; Pape et al., 2010). Current knowledge indicates that SA-promoted redox modification of Cys residues in NPR1 determines the levels of the active, reduced, and monomeric form of NPR1 in the nucleus (Kinkema et al., 2000; Mou et al., 2003; Tada et al., 2008; Lindermayr et al., 2010). The levels of nuclear NPR1 are also regulated by other SA-mediated mechanisms, such as proteasome-mediated degradation and phosphorylation (Pajerowska-Mukhtar et al., 2013).

Salicylic acid is essential for NPR1 redox modification, but how it controls this process is still not well understood. NPR1 reduction is catalyzed by thioredoxin TRXh5 (Tada et al., 2008; Kneeshaw et al., 2014), coded by the only member of *TRXh* gene class transcriptionally induced by SA and oxidative stress (Laloi et al., 2004; Tada et al., 2008; Belin et al., 2014). Whether NPR1 monomerization also occurs under oxidative stress, has not been explored yet. Furthermore, evidence indicates that both, oligomerization and monomerization of NPR1 involves *S-*nitrosoglutathione (GSNO) mediated *S*-nitrosylation (Feechan et al., 2005; Rusterucci et al., 2007; Lindermayr et al., 2010).

Non-Expressor of *PR* genes 1 reduction and therefore induction of NPR1-dependent genes, including *WRKYs* and *PR1*, correlate with the reductive phase of the defense response (Mou et al., 2003). Based on the evidence summarized here, we propose a model for SA-mediated NPR1 redox control and its influence on *PR1* induction (**Figure 2**, left panel).

Interestingly, the discovery of the direct binding of SA to NPR1 (Wu et al., 2012), and also to NPR3, and NPR4, which control NPR1 degradation (Fu et al., 2012), suggests the existence of a direct mechanism by which nuclear NPR1 levels and activity can be regulated according to the levels of SA, that in turn reflects the cellular redox state.

### TGA Factors, a Potential Node for Integrative Cellular Redox Regulation?

TGA factors have been postulated as redox sensors (Spoel and Loake, 2011), based on evidence showing that modification of Cys residues in TGA1 and TGA4 modulate their binding to NPR1 and to DNA (Despres et al., 2003; Lindermayr et al., 2010). TGA1 and TGA4 compose class I TGA and their function is not critical for the expression of SA-regulated genes (Kesarwani et al., 2007; Shearer et al., 2012; Wang and Fobert, 2013; Herrera-Vásquez et al., 2014). In contrast, the evidence supports that class II TGAs (TGA2, TGA5, and TGA6), and to a lesser extent TGA3, are the essential factors for SA-regulated expression of defense genes (Johnson et al., 2003; Zhang et al., 2003; Kesarwani et al., 2007; Herrera-Vásquez et al., 2014). Intriguingly, there is still no direct evidence of regulation of these factors through redox modification. Nevertheless, a potential for TGA2/5/6 as a node for general redox regulation in response to stress, is supported by the evidence described below.

TGA2 represses *PR1* basal expression but can also activate it upon SA-mediated stress challenge by interacting with negative and positive TGA boxes at the *PR1* promoter (Johnson et al., 2003; Zhang et al., 2003; Kesarwani et al., 2007; Pape et al., 2010). The essential role of TGA2/5/6 in *PR1* expression can be extrapolated to the group of NPR1-dependent genes with overrepresentation of the TGA box (Maleck et al., 2000).

We have shown that TGA2/5/6 are also essential for early SA-dependent and NPR1-independent induction of a set of genes with antioxidant and detoxifying activities (Blanco et al., 2009).* GRXC9*, which codes for a glutaredoxin of the plant-specific CC subfamily, is used here as a model for this pathway (**Figure 2**, medium panel; Ndamukong et al., 2007; Blanco et al., 2009; Herrera-Vásquez et al., 2014). SA-induced expression of *GRXC9* requires two *as-1* promoter elements that constitutively bind TGA2 and TGA3 factors (Herrera-Vásquez et al., 2014). *as-1* elements, consisting of two TGA boxes separated by four base pairs (Krawczyk et al., 2002), confer early and transient induction by SA through ROS (Qin et al., 1994; Johnson et al., 2001; Garreton et al., 2002). Two *as-1* elements were also found in the *TRXh5* promoter, although its functionality has not been explored yet (Laloi et al., 2004). We propose that early induction of *GRXC9*, and probably of *TRXh5* also, occurs during the oxidative phase of the defense response mediated by ROS signals (**Figure 2**; Mou et al., 2003; Herrera-Vásquez et al., 2014). TGA2/5/6 are also essential for the induction of *as-1*-containing genes involved in chemical detoxification (Mueller et al., 2008; Stotz et al., 2013).

Furthermore, the well-recognized antagonistic effect of SA on JA/ET-mediated transcriptional responses (Pieterse et al., 2009), is also mediated by class II TGAs (Ndamukong et al., 2007; Zander et al., 2010). SA inhibits expression of a group of JA/ET-induced genes, including *PDF1.2*, through repression of *ORA59*, which codes for a master transcription factor from the AP2/ERF family (Zander et al., 2010; Van der Does et al., 2013). Interestingly, ACC-induced and SA-repressed *ORA59* expression depend of TGAs class II factors, through their binding to a TGA box present in the *ORA59* promoter (**Figure 2**, right panel; Zander et al., 2014). Kinetic and pharmacological studies indicate that SA suppresses JA-responsive genes only within a specific time frame requiring SA-mediated increase in GSH levels (Koornneef et al., 2008). Therefore, SA-mediated *ORA59* repression occurs in the reductive phase of the defense response, which is consistent with evidence indicating that NPR1 is required for SA-mediated repression of JA/ET-induced genes (Spoel, 2003).

Taken together, we can conclude that class II TGAs (particularly TGA2) are essential in different mechanisms of transcriptional control mediated by SA and ROS signals, which operate at different times in the defense response to stress (**Figure 2**). Accordingly, a strong phenotype of stress sensitivity is detected in *tga2/5/6* triple mutant plants (Zhang et al., 2003; Mueller et al., 2008). The question is how TGA2 activity is controlled by SA and ROS signals? The only clue for a redox control of TGA2 is that it interacts with GRXC9 in the nucleus (Ndamukong et al., 2007). Interestingly, *GRXC9* overexpression represses the expression of its own gene and of *ORA59* while GRXC9 forms part of the complex bound to the *as-1*-containing region of the *GRXC9* promoter (Herrera-Vásquez et al., 2014; Zander et al., 2014). These findings are integrated in the model shown in **Figure 2**. This model shows that SA, by inducing expression of *GRXC9*, controls the expression of antioxidant genes and at the same time represses JA/ET-mediated responses. We speculate that GRXC9 catalyzes the reduction of a protein from the transactivating complex in both genes, triggering their inactivation. Although evidence for functional associations of TGA factors and CC-type GRXs suggests that TGAs can be redox-modified (Murmu et al., 2010), there is still no evidence of this modification.

### Oxidoreductases as Redox Sensors in the SA-Mediated Control of Gene Expression

The involvement of TRX/GRX oxidoreductases in SA-mediated transcription was first proposed some years ago (Fobert and Despres, 2005). As described above, two Cys-containing oxidoreductases, TRXh5, and GRXC9, were later on recognized as important elements for redox control in SA-mediated transcriptional responses. *TRXh5* and *GRXC9* genes are induced by SA during the oxidative phase of the defense response. TRXh5 reduces NPR1, which is essential for NPR1-dependent transcriptional responses (Tada et al., 2008). Instead, GRXC9 probably reduces a still unknown protein that represses the expression of genes from SA-dependent NPR1-independent as well as JA/ET-dependent SA-repressed pathways. These processes occur during the reductive phase of the defense response (**Figure 2**). Considering that TRXh5 and GRXC9 are in turn reduced and regenerated at the expense of the reducing power of NADPH and GSH, respectively (Meyer et al., 2012), these enzymes become key redox sensors that coordinate transcription and the cellular redox state.

## Conclusion and Future Directions

The evidence discussed here indicates that redox-modulated processes are critical for the fine-tune regulation of gene expression mediated by SA. These processes occur in a temporaly controlled manner, coordinated with the cellular redox changes occurring during the defense response. Although important advances have occurred during the last years, we still have a fragmented knowledge of the network of redox processes that allows a coordinated transcriptional response to stressful conditions. Focusing on SA–ROS interplay, one important challenge is to understand how ROS generated in different cell compartments and cell types triggers SA biosynthesis. Furthermore, considering that all stress conditions generate oxidative bursts, but not all lead to SA accumulation, how is the specificity of ROS signals for triggering SA biosynthesis established? A point of convergence of the responses to different stresses mediated by SA, such as the PAD4/EDS1/SAG101 complex located upstream in the SA-signaling pathway (Wiermer et al., 2005), can be explored as a node for redox regulation of SA biosynthesis in response to stress.

In relation to the redox mechanisms that control the SA-mediated transcriptional response, the evidence discussed here supports the involvement of NPR1, TGA factors, and the oxidoreductases TRXh5 and GRXC9 as redox sensors. Several intriguing aspects about these sensors are pending, such as the promiscuous and essential role of TGA2 in the control of genes that respond to oxidant and reducing cellular redox states. Whether TGA2 itself can be redox modified, particularly reduced by GRXC9 to trigger gene repression during the reductive phase of the defense response, is a critical point that still needs to be answered. In this context, an interesting target to explore for redox regulation is MED18. The MED18 protein is a member of the Mediator Complex that interacts with the Ying Yang 1 transcription factor (YY1; Lai et al., 2014). This complex co-represses three genes coding for oxidoreductases involved in defense: GRXC9, TRXh5, and GRXS13 (La Camera et al., 2011; Laporte et al., 2012).

Finally, an important challenge for the future is to incorporate the temporal and spatial perspective in the analysis of the redox processes associated to transcriptional activity. New technical approaches that allow to record cell-specific changes in ROS levels, the redox state of GSH and new markers for gene expression will help in unraveling the sequential events occurring in different groups of cells exposed to stress during the time course of the defense response.

## Conflict of Interest Statement

The authors declare that the research was conducted in the absence of any commercial or financial relationships that could be construed as a potential conflict of interest.
