# Corrigendum: Salicylic acid and reactive oxygen species interplay in the transcriptional control of defense genes expression

**DOI:** 10.3389/fpls.2017.00964

**Published:** 2017-05-31

**Authors:** Ariel Herrera-Vásquez, Paula Salinas, Loreto Holuigue

**Affiliations:** Departamento de Genética Molecular y Microbiología, Facultad de Ciencias Biológicas, Pontificia Universidad Católica de ChileSantiago, Chile

**Keywords:** glutathione, glutaredoxin GRXC9/GRX480, NPR1, reactive oxygen species, salicylic acid, thioredoxin TRXh5, TGA transcription factors

In the original article, we neglected to include the funder **National Commission for Science and Technology CONICYT, FONDECYT grant N° 1141029**. The authors apologize for this error and state that this does not change the scientific conclusions of the article in any way.

## Conflict of interest statement

The authors declare that the research was conducted in the absence of any commercial or financial relationships that could be construed as a potential conflict of interest.

